# Dog Bite to the Hand: Infectious Disease Considerations

**DOI:** 10.7759/cureus.65964

**Published:** 2024-08-01

**Authors:** Sophia Taylor, Nofel Iftikhar, Kevin M Sherin, Latha Ganti

**Affiliations:** 1 Biology, Boston University, Boston, USA; 2 Biology, University of Florida, Gainesville, USA; 3 Primary Care, Orlando College of Osteopathic Medicine, Winter Garden, USA; 4 Emergency Medicine & Neurology, University of Central Florida, Orlando, USA; 5 Research, Orlando College of Osteopathic Medicine, Winter Garden, USA; 6 Medical Science, The Warren Alpert Medical School of Brown University, Providence, USA

**Keywords:** stray dog, cellulitis, tetanus vaccine, rabies vaccine, dog bite

## Abstract

We present the case of a 27-year-old man who sustained a bite wound from a stray dog found on the side of a highway. He had cleaned the wound well, hoping to avoid infection, but when it swelled and became red, he sought medical attention. The authors describe the management of cellulitis and prophylaxis for rabies and tetanus.

## Introduction

The category of "animal attacks" can include several potential injuries. The most dangerous, however, are penetrating bites due to the added risk of infection that can be detrimental to the victim. Among animal bites, dog bites are the most common, accounting for 4.5 million cases every year [1]. Dog bites are most likely to occur from a canine familiar to the victim, such as a domesticated pet, as opposed to a dog with no relation to an owner, like a stray as in this case. Children are the most at risk for dog bites and most commonly sustain injuries in their head or neck [2]. In adults, the most common area of injury is the hand, where infection is also most likely to occur [1]. Factors that may compound the risk of infection in the injury include the severity of the bite and immediate disinfection and wound care for the victim. Although minor punctures can heal independently, symptoms such as redness, swelling, and persistent pain indicate infection [3]. In addition to cellulitis, tetanus and rabies are the most feared infections resulting from dog bites. 

## Case presentation

**Figure 1:**
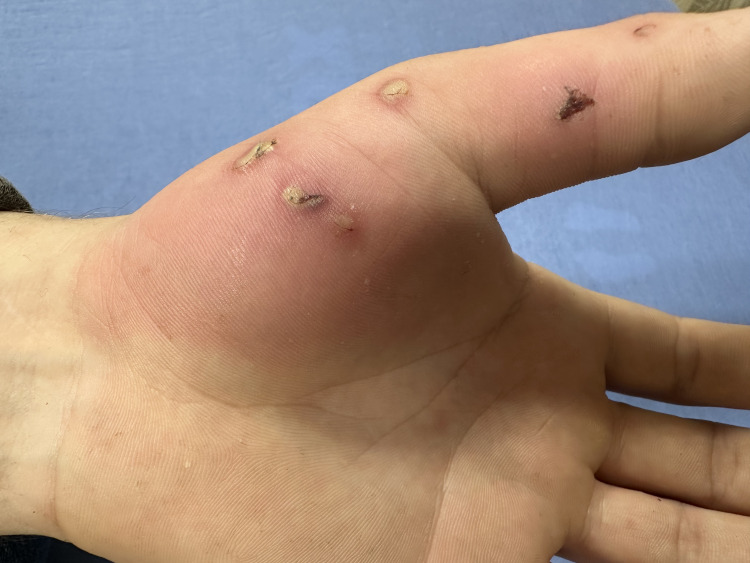
Photograph of the patient's left hand depicting puncture wounds and surrounding cellulitis

A 27-year-old male presented to the emergency department (ED) in the Southeastern United States having sustained a dog bite two days prior. The man encountered a stray dog on the side of the highway. Hoping to help it and take it home, the man approached but was promptly bitten, sustaining multiple puncture wounds on his left hand, centralized around the thumb. He immediately drove to a gas station to obtain alcohol and peroxide to disinfect the site. The man presented to the ED due to redness in the area and the concern for potential infection compounded by a history of heavy smoking. His vital signs were as follows: oxygen saturation 97% on room air, blood pressure 140/93 mmHg, mean arterial pressure 108 mmHg, temperature 97.8 degrees Fahrenheit, pulse 88 beats per minute, and respiration rate 18 breaths per minute. The bite wounds were minor, circular, and deep, although no pus was present, only redness in the area that appeared to have reduced since the initial attack (Figure 1).

Following a physical examination, the affected area was determined to be cellulitis. The patient was given Augmentin 875 mg, for 10 days to be taken twice daily. Due to the dog being a stray with no known vaccination or health history, the patient was given prophylaxis for rabies and tetanus. He received the first rabies vaccine and toxoid dose in the ED and would return for the rest of the vaccine series on days 3, 7, and 14, as he had not been previously vaccinated against rabies. He also received the intramuscular tetanus toxoid. The man was sent home and informed to return to the ED if the area of redness spread.

## Discussion

Cellulitis is a bacterial infection affecting the dermis and subcutaneous tissues, causing tenderness, redness, and swelling [3]. The infection is most commonly the result of *Streptococcus* and *Staphylococcus* bacteria being introduced to the skin microbiome; however, cellulitis from traumatic scratches or animal bites is most commonly caused by *Pasteurella multocida* [4]. A bacterium particular to dog bites is *Capnocytophaga canimorsus*.

Treatment for acute cellulitis includes oral antimicrobials, most commonly against *Staphylococcus aureus*, though treatments may vary depending on accessibility and patient allergy profile. If the infection spreads, intravenous intervention may be necessary. In more severe cases, surgery should be considered for washout and to debride necrotic tissue [3].

The described patient was at an increased risk for infection due to his history of smoking. Tobacco use is associated with altered respiratory function and compromised immune response, which, naturally, increases susceptibility to infection and increases recovery time following infection [5].

Rabies is a neglected tropical disease that [1] causes tens of thousands of deaths annually, with 40% being children under age 15 [6]. The history of rabies in America dates to the 18th century and the Columbian exchange, with European colonizers transporting infected animals from Europe to America. The proliferation of the disease in the Americas was slowly curbed by Pasteur's development of the rabies vaccine in 1885. Following the development of Pasteur's initial vaccine, several other subsequent vaccines, and regulations mandating pet, wildlife, and livestock vaccination against rabies, rates of the disease, in both animals and humans, plummeted, evidenced by the low observance of the disease in the modern day [7-8]. In the United States, dog-mediated rabies, and pet-mediated rabies in general, has been largely extinguished since the aforementioned implementation of mass vaccination practices for pets. Therefore, in the United States, rabies transmission most commonly occurs through bats, coyotes, foxes, raccoons, mongoose, and skunks. However, even in the United States, regional differences are present. For example, people living in Florida are more likely to get rabies from a fox versus someone living in New York, where bat-mediated rabies infection is more prevalent [9-10].

While within the United States, rabies infections from dogs, cats, and other household pets are relatively uncommon, in other countries, dog bites and scratches represent the most common means of transmission for rabies. This is most often the case in countries without mass vaccination programs for dogs or well-developed veterinary medicine infrastructure. India, for example, has one of the highest frequencies of rabies cases globally, the majority of which being mediated via stray dog bites [11].

While the rabies infection initially presents with nonspecific symptoms similar to a flu-like illness, once it reaches the central nervous system (CNS), symptoms progress to pathognomonic aerophobia, hydrophobia, agitation, confusion, and hallucinations. The incubation period for the disease from the infected site to the CNS can last from weeks to months; thus, taking a good history of present illness is important. Once present in the CNS, and clinical symptoms appear, rabies is fatal in 100% of cases [12-13].

The majority (80%) of patients who contract rabies present with classic "furious symptoms," which include hyperactivity, excitable behavior, hydrophobia (fear of water), and sometimes aerophobia (fear of drafts or of fresh air). The remaining 20% may present with paralytic symptoms, which include weakness, sensation alteration, incontinence, and myoedema [14]. The location and severity of the bite affect the speed at which the disease takes root within the body and, thus, the signs and symptoms present.

**Figure 2:**
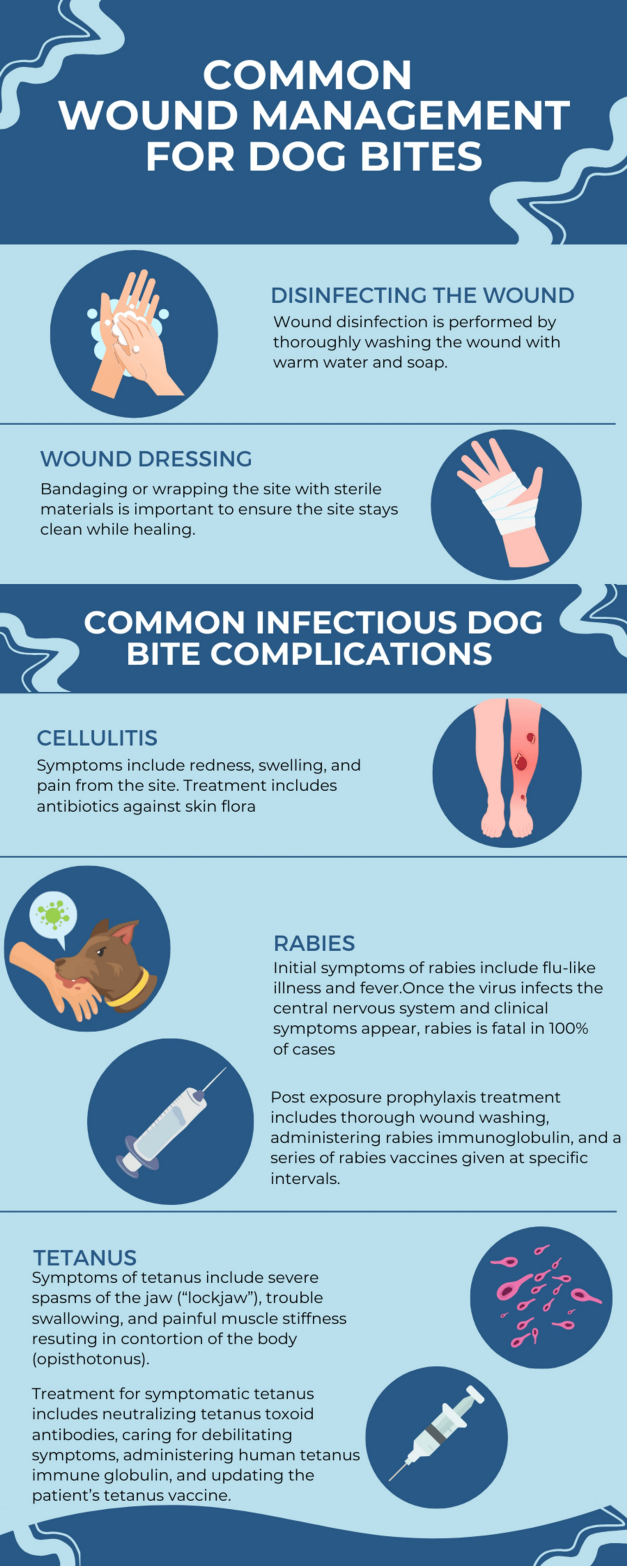
Infographic describing common wound management for dog bites Image Credit: Sophia Taylor on canva.com

Since the patient was bitten by a dog with unknown medical history and which could be a carrier of rabies, the Centers for Disease Control and Prevention recommendations would include both immunoglobulin and rabies vaccination [15-16]. The general protocol for rabies management calls for a four-series vaccine for persons not previously vaccinated against rabies (such as our patient), whereas someone previously vaccinated would only receive two vaccinations [15] (Figure 2).

The immunoglobulin is administered directly into the wound to ensure passive immunity remains intact after the initial dose of the rabies vaccine. The recommended dose of rabies immunoglobulin is 20 IU/kg of body weight. If not given at day 0, the immunoglobulin can be administered up to day 7; after this, it can be assumed that the body has already developed antibodies and the immunoglobulin would be insufficient. The vaccine is given in the shoulders of adults, and children's thighs, and doses are repeated on days 3, 7, 14, and 28 [16]. Prompt intervention and post-exposure prophylaxis (PEP) are crucial for increasing the chance of survival from rabies.

After the ED's care, PEP for completing the rabies vaccine doses may be limited depending on access to care [16]. Public health departments may assist with PEP, animal quarantine, animal brain analysis, or public notification of suspected rabid animals [17].

In addition to rabies, tetanus represents another potential infectious complication resulting from animal bites. Tetanus is caused by a bacterium called *Clostridium tetani*, which is relatively common in everyday flora and fauna [18]. Tetanus is transmitted to humans through penetrative wounds from an infected animal or object. The incubation time varies from 24 hours to multiple months, depending on the severity of the wound and the quantity of bacteria transmitted [19]. Symptoms of the disease include painful, uncontrollable spasms. These spasms progressively become more intense, leading to trouble breathing and eventually death [18].

Tetanus cases around the world have dramatically decreased due to access to vaccinations. In the United States, only 0.01 per 100,000 people contract tetanus [16]. This number is much higher in developing countries due to limited access to vaccinations and treatments. Without proper medical treatment, the fatality rate is near 100% [19]. Tetanus treatment includes steps to neutralize the tetanus toxoid antibodies, providing care for symptoms, like spasms and respiratory weakness, and possibly administering human tetanus immunoglobulin. With proper treatment, the fatality rate for tetanus drops to around 20% [20].

## Conclusions

Dog bite wounds, though seemingly harmless, are serious injuries that often require prompt medical attention for wound care. These precautions include treating minor infections and, more importantly, preventing rabies and tetanus to improve patient outcomes and recovery.

## References

[1] Bula-Rudas FJ, Olcott JL (2018). Human and animal bites. Pediatr Rev.

[2] Desai AN (2020). Dog bites. JAMA.

[3] Sullivan T, de Barra E (2018). Diagnosis and management of cellulitis. Clin Med (Lond).

[4] Pithadia DJ, Weathers EN, Colombo RE, Baer SL (2019). Severe and progressive cellulitis caused by Serratia marcescens following a dog scratch. J Investig Med High Impact Case Rep.

[5] Arcavi L, Benowitz NL (2004). Cigarette smoking and infection. Arch Intern Med.

[6] (2024). Rabies. https://www.who.int/news-room/fact-sheets/detail/rabies.

[7] Velasco-Villa A, Mauldin MR, Shi M (2017). The history of rabies in the Western Hemisphere. Antiviral Res.

[8] Liu C, Cahill JD (2020). Epidemiology of rabies and current US vaccine guidelines. R I Med J.

[9] O'Sullivan B, Burke R, Bassaline D (2019). Notes from the field: rabies exposures from fox bites and challenges to completing postexposure prophylaxis after hurricane Irma - Palm Beach County, Florida, August-September 2017. MMWR Morb Mortal Wkly Rep.

[10] Chang HG, Eidson M, Noonan-Toly C (2002). Public health impact of reemergence of rabies, New York. Emerg Infect Dis.

[11] Radhakrishnan S, Vanak AT, Nouvellet P, Donnelly CA (2020). Rabies as a public health concern in India-a historical perspective. Trop Med Infect Dis.

[12] Bampoe VD, Brown N, Deng L (2024). Serologic immunity to tetanus in the United States, National Health and Nutrition Examination Survey, 2015-2016. Clin Infect Dis.

[13] Bastos V, Pacheco V, Rodrigues ÉD (2023). Neuroimmunology of rabies: new insights into an ancient disease. J Med Virol.

[14] Hemachudha T, Laothamatas J, Rupprecht CE (2002). Human rabies: a disease of complex neuropathogenetic mechanisms and diagnostic challenges. Lancet Neurol.

[15] Arsuaga M, de Miguel Buckley R, Díaz-Menéndez M (2024). Rabies: epidemiological update and pre- and post-exposure management. Med Clin (Barc).

[16] (2024). Rabies VIS. https://www.cdc.gov/vaccines/hcp/vis/vis-statements/rabies.html.

[17] (2024). Control of neglected tropical diseases. https://www.who.int/teams/control-of-neglected-tropical-diseases/rabies/vaccinations-and-immunization.

[18] Howington GT, Nguyen HB, Bookstaver PB, Akpunonu P, Swan JT (2021). Rabies postexposure prophylaxis in the United States: opportunities to improve access, coordination, and delivery. PLoS Negl Trop Dis.

[19] Farrar JJ, Yen LM, Cook T, Fairweather N, Binh N, Parry J, Parry CM (2020). Tetanus. J Neurol Neurosurg Psychiatry.

[20] (2024). Tetanus. https://www.who.int/health-topics/tetanus.

